# Paradoxical Facilitation of Attention in Healthy Humans

**DOI:** 10.1155/2006/632141

**Published:** 2006-11-27

**Authors:** Shirley Fecteau, Alvaro Pascual-Leone, Hugo Théoret

**Affiliations:** ^1^Center for Non Invasive Brain StimulationBeth Israel Deaconess Medical CenterHarvard Medical SchoolBostonUSA; ^2^Département de psychologie and Centre Hospitalier Universitaire Sainte-JustineUniversité de MontréalCanada

## Abstract

Transcranial magnetic stimulation (TMS)-induced virtual lesions in healthy subjects can be used to test neurofunctional models of disease. The interhemispheric rivalry model of heminglect is well suited for such investigations, as simple predictions derived from clinical data can be tested without the caveats normally associated with lesion studies. One of these predictions is that release from contralateral inhibition should lead to increased parietal responsiveness, which, in turn, would enhance spatial attention. Here, we detail studies showing TMS-induced paradoxical functional facilitation of attention in healthy individuals and highlight their contribution to the understanding and treatment of neglect syndromes.

## 1. Introduction

Cerebral hemispheres are continuously interacting through inhibitory and excitatory influences. One classic model of interhemispheric interaction is the one of rivalry, in which hemispheres have mutual, reciprocal inhibitory influences [4]. As detailed in this issue, the interhemispheric competition model of attention has been the basis for novel approaches aimed at understanding and treating neglect syndromes. It has been proposed that neglect is related to an imbalance between the hemispheres resulting from the release of reciprocal inhibitory influences. In much the same way that a second lesion in the opposite hemisphere resulted in recovery from neglect in a now famous patient [17], it has been hypothesized that inducing a virtual brain lesion to disrupt brain activity in the homologous region of the contralesional hemisphere would lead to behavioral improvement. Such recovery has indeed been reported in neglect patients treated with TMS, providing experimental support for the original hypothesis.

TMS is a powerful tool in the arsenal of cognitive neuroscientists because of its ability to establish causal relationships between brain and behavior in healthy individuals [11]. In attention, TMS finds an ideal application because it can be used to verify specific hypotheses derived from clinical data in both lesioned and normal populations. In patients with visual neglect, the healthy hemisphere appears to perform better-than-normal when interhemispheric rivalry is disrupted after a stroke [6,14]. Following on these data and theoretical suggestions, two simple predictions of the interhemispheric competiton model of attention can be made: $i$) TMS (single, paired or repetitive) over the contralesional hemisphere in neglect patients will reduce attentional deficits; and *ii*) TMS (single, paired or repetitive) over the parietal cortex of normal subjects will lead to enhanced performance on the ipsilateral side.

The first prediction has been tested in neglect patients (see Oliveri et al. and Fierro et al., this issue). It has been shown, for instance, that application of single TMS pulses to the contralesional frontal cortex reduces the rate of contralateral extinction in the tactile modality [7] and that repeated TMS over the unaffected parietal cortex reduces patient errors on the line bisection task [8]. As such, the interhemispheric rivalry model has recently garnered much support in neglect patients. The second prediction, stating that paradoxical facilitation of attentional processes should occur following TMS-induced virtual lesions, has been tested in neuro-

ISSN 0953-4180/06/$17.00 © 2006 – IOS Press and the authors. All rights reserved logically intact subjects. The underlying hypothesis is that TMS over the parietal cortex should result in trans-hemispheric release of inhibition in the contralateral hemisphere, which in turn would become hyperactive and create hyper-attention to the ipsilateral side. Seyal and colleagues [12] were first to demonstrate perceptual paradoxical facilitation in healthy subjects by showing increased sensitivity to cutaneous stimuli following TMS over the parietal lobe. Fifty msec after a single TMS pulse was applied to right parietal lobe, an electrical stimulus was applied to the right thumb. Participants were instructed to report the presence or absence of the thumb stimulus after each trial. It was found that the number of perceived stimuli increased following TMS of the parietal lobe compared to frontal TMS and no-TMS conditions. In addition, the ipsilateral sensory threshold was significantly reduced following parietal TMS, whereas it was unchanged following frontal or sensorimotor TMS.

This first study highlights the contribution that virtual-lesion approaches can make to our understanding of normal and pathological brain processes. The use of healthy subjects, in which brain mechanisms are only transiently disrupted, allows within-subject comparisons with baseline performance. This is a real advantage because in addition to less than systematic lesion locations, pre-lesion performance in brain-damaged patients is rarely available, making it hard to establish rigourous performance criteria. A second demonstration of TMS-induced paradoxical facilitation of attention took advantage of this test-retest possibility. Hilgetag and collaborators [3] used 'off-line' TMS [11] to probe ipsilateral performance enhancements in visual attention. In off-line paradigms, magnetic stimulation and behavior are dissociated in time. This is based on the fact that when applied repetitively (rTMS; at a low frequency of 1 Hz), TMS can reduce cortical excitability beyond the train of stimulation [2]. This design enables comparison of perfomance pre- and post-TMS within the same subject.

In the Hilgetag study, the effects of parietal rTMS were evaluated on a simple visual detection task. Stimulus size was carefully selected for each subject individually to account for inter-individual differences in attentional capabilities. Baseline performance was first established, where participants had to detect small rectangular stimuli briefly presented either unilaterally in the left or right peripheral visual field, or bilaterally in both. Detection peformance was also tested immediately after a ten minute rTMS train to (a) right parietal cortex; (b) left parietal cortex; (c) right primary motor cortex; and (d) sham stimulation. Magnetic stimulation targeted the intraparietal sulcus although rTMS presumably disrupts a somewhat non-focal area of cortex. It was found that participants could detect significantly more targets in the visual field ipsilateral to the stimulated hemisphere following rTMS to the parietal cortex, whether it was applied to the left or right hemisphere. This paradoxical increase in performance was accompanied by a decreased performance for stimuli presented contralaterally to the stimulated hemisphere. None of the control stimulation sites had any effect on detection performance, confirming the specificity of the effect to the parietal cortex. The presence of both increases and decreases in performance makes it unlikely that the effects were solely perceptual in nature. Comparisons within the same visual field, but following different TMS locations, show that ipsilateral performance is indeed increased and independent of the contralateral effect, in addition to comparing ipsilateral performance pre- and post-rTMS. Again here as in the Seyal et al. study, a virtual lesion design in healthy participants allowed for a direct verification of the interhemispheric competition model of attention. In addition to the demonstration of increased ipsilateral performance, a second prediction of the model was verified: that contralateral performance should decrease following inactivation of parietal cortical areas. One possible neural mechanism for these effects may be that TMS transiently reduces excitability of mainly inhibitory transcallosal fibers. This, in turn, would release inhibitory mechanisms within the contralateral hemisphere. It goes without saying that the exact mechanism by which inhibition is released transcallosally needs to be addressed with greater detail to truly understand interhemispheric competition and its impact on normal and pathological behavior.

What is clear from the aforementionned studies is that in ideal conditions, TMS in healthy individuals can lead to what has been termed 'virtual neurology' [10]. This is nowhere more evident than in situations where specific predictions regarding brain organization, and the effects of lesions on behavior, are available. In the case of paradoxical facilitation and its underlying hypothesis of interhemispheric competition, the 'ideal conditions' are present. First, there exists a specific, testable hypothesis of functional brain organization [4]. Second, behavioral data from lesion patients support the hypothesis of increased ipsilesional performance in neglect patients [6,14]. Third, TMS data from normal individuals closely mirror the deficits and enhancements reported in patients [3,9,12]. Finally, TMS can be used to test the original hypothesis in lesion patients and ultimately provide therapeutic avenues [1,7].

Another interesting question raised by TMS investigations of paradoxical facilitation is whether hemispheric rivalry is a brain organizing principle that could be applied to other aspects of human behavior. Of particular relevance is the fact that patients with strokes involving the primary motor cortex (M1) often display increased excitability of the contralateral M1 [13,16]. This is reminiscent of what is described in neglect patients and, as such, inhibition of M1 in healthy individuals could lead to ipsilateral motor improvement through the release of contralateral inhibiton. Kobayashi and collaborators [5] applied low-frequency (1 Hz) rTMS over the primary motor cortex to test the interhemsipheric competition hypothesis. Performance on a sequential key-pressing task was evaluated pre- and post-rTMS. Suppression of motor cortex excitability significantly reduced execution time of the motor task performed with the ipsilateral hand. As in the studies of attention, stimulation of control sites did not affect performance. In strikingly similar fashion to what has been reported in neglect syndromes, repeated TMS was used in stroke patients to determine whether downregulation of contralesional motor cortex could improve motor function within the affected hand [15]. Slow-frequency rTMS applied for 25 minutes over the contralesional primary motor cortex resulted in a significant increase in pinch acceleration of the affected hand, compared to sham stimulation. These results suggest that paradoxical functional facilitation is not restricted to attentional processes, but can also occur in response to disruption of cortical areas outside parietal cortex. It bears mention that the issue of therapeutic effect duration of slow-frequency rTMS is far from resolved. Differences in stimulation parameters, stimulated areas and disease make it hard to establish a clear timeframe for how long the benefits of rTMS last. It is evident that future studies need to address this question directly if rTMS is to become a standard treatment approach.

Taken together, the reviewed studies showing paradoxical functional facilitation with TMS in healthy subjects support predictions stemming from the interhemispheric rivalry hypothesis of brain function. Specifically, neurodisruption of a focal brain area appears to induce contralateral disinhibition in homologous areas, which, in turn, increases performance ipsilaterally to the stimulation site. It is important to note that such behavioral improvements can come with a cost: whereas spatial attention is increased in the ipsilateral visual field following a TMS-induced virtual lesion to the parietal cortex, performance in the contralateral field is reduced [3]. This underscores the notion that whatever brain effects are being produced by TMS, they are not occuring in isolation. Rather, TMS targets a specific node of a presumably distributed neural network underlying the behavior of interest. In that sense, the increases and decreases in behavioral output that are created by TMS can be better explained in terms of the consequences of how the brain deals with downregulation of a particular area.

In conclusion, despite these caveats, the functional changes that are transiently induced by neurodisruption of the healthy human brain are providing crucial knowledge regarding normal interhemispheric balance, as well as brain plasticity following stroke. These findings underscore the potential of TMS-induced virtual lesions to generate behavioral improvements. Of particular relevance is the fact that data in normal subjects can provide valuable insight into plastic and pathological changes that occur following a stroke. In that sense, studies of paradoxical functional facilitation in normal subjects have contributed a great deal to the development of novel therapeutic strategies for stroke patients. As must be clear from this special issue, we are only beginning to determine the possibilities afforded by neuromodulation in normal and diseased individuals.
